# Effects of an external magnetic field on microbial functional genes and metabolism of activated sludge based on metagenomic sequencing

**DOI:** 10.1038/s41598-020-65795-3

**Published:** 2020-06-01

**Authors:** Shuying Geng, Weizhang Fu, Weifeng Chen, Shulian Zheng, Qi Gao, Jing Wang, Xiaohong Ge

**Affiliations:** 1 College of Resources and Environment, Shandong Agricultural University, Taian, 271018 China; 2 College of Water Sciences, Beijing Normal University, Beijing, 100875 China; 3 Taian Chuanyuan Environmental Protection Equipment Co., Ltd, Taian, 271000 China; 4 College of Food Science and Engineering, Shandong Agriculture and Engineering University, Dezhou, 251100 China

**Keywords:** Environmental sciences, Enzymes

## Abstract

This study explored the effect of 70-mT magnetic field on wastewater treatment capacity for activated sludge in long-term laboratory-scale experiments. Metagenomic sequencing were conducted based on Illumina HiSeq 2000 platform after DNA extraction of the activated sludge. Then the effect of the magnetic field on the microbial unigene and metabolic pathways in activated sludge was investigated. As a result, higher pollutant removal was observed at 70 mT, with which the elimination of total nitrogen (TN) was the most effective. Functional genes annotated based on eggNOG database showed that unigenes related to information storage and processing were enhanced by the magnetic field. For CAZy classification, category such as glycosyl transferases was more abundant in the reactor with magnetic field, which has been shown to promote the entire energy supply pathway. Additionally, in the KEGG categories, unigenes related to signaling molecules and interaction were significantly inhibited. Through the enrichment analysis of the nitrogen metabolism pathway, the magnetic field inhibited anabolic nitrate reduction by significantly inhibiting enzymes such as [EC:1.7.7.2], [EC:1.7.7.1], [EC:3.5.5.1], [EC:1.4.1.2] and [EC:4.2.1.1], which are related to the improvement of the denitrification ability. This study can provide insight for future research on the response mechanism of activated sludge to magnetic fields.

## Introduction

New methods to improve the effect of the wastewater treatment have been sought by researchers. With the application of magnetic fields in environmental engineering to improve the processing systems or processes, the combination of the magnetic field and wastewater treatment technology has been subjected to increasing concerns. The magnetic field is applied to the crystallization of calcium carbonate, water purification, colloid condensation and precipitation, wastewater treatment, etc.^1^. The application of the magnetic field mainly improves the physical properties of solid-liquid separation by the aggregation of colloidal particles and the biological characteristics by improving the activity of bacteria, to improve the performance of wastewater treatment^2^.

Since 1975, when American scientist Blakemorels first discovered magnetotactic bacteria^3^, an increasing number of researchers have studied the effects of magnetic fields on microorganisms. Meanwhile, the definition of the magnetic biological effect has been gradually formed^4^. In fact, different magnetic field types and intensities, action times and biological cell structures cause different biological effects, which may be promoted or inhibited ^5,6^. The magnetic field has different effects on the cell wall transport mechanism of gram-positive and gram-negative bacteria^7^. However, the impact of magnetic fields on biological systems has been poorly studied^8^.

At present, the application of magnetic technology mainly includes permanent magnets, high-gradient magnetic separation, magnetic adsorption or electromagnetic devices^2^. Different ways of magnetic applications have different effects on the performance of each wastewater treatment system. According to Yavuz *et al*., experiments operating at direct current (DC), pulsed DC and alternating current (AC) magnetic field exhibited that AC magnetic field has a positive effect on the removal efficiency. And application of magnetic field also increased the microorganism’s growth rate up to 17.8 mT and then decreased^9^. Others using magnetostatic device have found that the growth of activated sludge biomass and dehydrogenase activity positively affected by a 7-mT magnetic field. Moreover, under this magnetic field, compared with the control group, the reduction rate of COD (chemical oxygen demand) in magnetic-field-exposed samples increased by 26%^10^. For the method of adding permanent magnets used in this study, most of the relevant studies^11,12^ use magnetic field intensity greater than 10 mT. Our pre-experiments and previous studies on the external magnetic field at different intensities (70–150mT) confirmed that 70 mT has the most obvious improvement on the wastewater treatment performance and has significant influence on the activated sludge microbial community^13^.

Microorganisms in activated sludge play a key role in the degradation of pollutants. However, the effect of the magnetic field on the microorganism functional gene and metabolism of activated sludge remains rare in the literature reviews. Studies found that under the condition of magnetic field exposure, a 5-mT magnetic field could enhance the AOB activity of the PN bacterial community and decrease the bacterial diversity. Compared with no external magnetic field and high external magnetic field, the expression of functional genes related to the microbial signal transduction, cell viability and signal transduction in the environment of 5 mT was higher^14^. In fact, the structural stability and diversity of these microbial communities are closely related to the biological treatment effect. Therefore, to fully reveal the mechanism of the magnetic field in improving the wastewater treatment performance, it is necessary to deeply study the microbial community structure and function in activated sludge.

In addition, for the research on microbial diversity and function, metagenomic sequencing i.e., whole genome sequencing of the total DNA extracted from the environment, is now widely used^15–17^. This method has been widely used to explore the taxonomic and functional diversity of microbial communities, which avoids the disadvantages of traditional cultivation-based methods. Reports on the application of metagenomic sequencing to study the microorganisms in activated sludge have revealed the high diversity of microbial species and functional genes in activated sludge^18,19^.

In this study, the long-term effect of magnetic field of induction 70mT on sequencing batch reactors (SBRs) was investigated. Two SBRs with (M) and without (CK) an external magnetic field were started up with identical running conditions. The performance of pollutants was evaluated at the ambient temperature (varying from 16.7°C to 28.3°C). Furthermore, the effect of the magnetic field on the microbial functional gene and metabolic pathways in activated sludge was studied by using the metagenomic high-throughput sequencing technology. The microbial function and metabolic pathways of the two reactors with and without magnetic field were compared. Thus, the magnetogenic effect was revealed at the gene level.

## Materials and Methods

### Inoculated sludge and synthetic wastewater

The inoculated sludge was the backflow sludge of a wastewater treatment plant (WWTP) in Tai’an City, Shandong Province, China. The volatile suspended solid (VSS) of the experimental SBRs after inoculation was 2.60 g·L^−1^ and 2.55 g·L^−1^ in CK and M, respectively. The composition of the synthetic wastewater was as follows: C_12_H_22_O_11_ (400 mg·L^−1^), (NH_4_)_2_SO_4_ (30 mg·L^−1^), MnSO_4_.H_2_O (2.68 mg·L^−1^), MgSO_4_.7H_2_O (180 mg·L^−1^), FeCl_3_.6H_2_O (0.134 mg·L^−1^), CaCl_2_ (10.6 mg·L^−1^), KH_2_PO_4_ (41 mg·L^−1^), K_2_HPO_4_ (28 mg·L^−1^)and 1.0 mL trace element solution according to Kong *et al*.^20^.

### Reactors setup and operation

Two parallel SBRs were operated at ambient temperature under seasonal change from May to September (125 days). The working volume of each reactor was 10.0 L. A pair of parallel permanent magnets (15 cm×10 cm×2.5 cm) was added to the SBR reactor to provide a 70-mT magnetic field. The treatment of the magnetic field was always continuous. The magnitude of the magnetic induction was adjusted by the distance. The reactors without the installation of permanent magnets and with 70-mT magnetic induction are denoted as CK and M, respectively. Oxygen was provided by an aeration pump, and the aeration volume was controlled by the valve on the aeration pipe. The operation stage of each cycle includes five stages: inlet, aeration, precipitation, drainage and idle period. The hydraulic retention time (HRT) was 12 h.

### DNA extraction and high-throughput sequencing

E.Z.N.A soil DNA kits (Omega Bio-Tek, Norcross, GA, USA) were used to extract the DNA from mixed activated sludge samples in long-term reactors. Based on the Illumina HiSeq. 2000 platform, the whole genome shotgun (WGS) strategy was used to randomly interrupt the extracted macrogenome total DNA into short fragments and construct the appropriate length of the inserted fragment libraries. Paired-end (PE) sequencing was performed on these libraries.

### Data quality control and sequence assembly

FastQC (http://www.bioinformatics.babraham.ac.uk/projects/fastqc/) was used for quality control on the raw data from sequencing. The total raw reads from sequencing were preprocessed to remove low-quality reads, such as those that are too short, contain too many fuzzy bases, or are incorporated into Adapter or host genomes. Then, the filtered clean reads were corrected using SOAPec (v2.01). According to the parameter setting of K-mer=57, SOAPdenovo2 (http://soap.genomics.org.cn/soapdenovo.html)^21^ was used for the de novo assembly of PE sequences in each sample. Contigs/scaffolds sequences were constructed by De Bruijn graph.

### Macrogenomic gene prediction and functional annotation

#### Genetic prediction and elimination of redundancy

Scaffold/Scaftig sequences of no less than 300 bp were selected from each sample, MetaGeneMark^22^ was used to conduct genetic prediction, and open reading frame (ORF) were recognized to obtain the corresponding protein sequences. Subsequently, CD-HIT^23^ was used to merge and remove the redundancy of these protein sequences according to 90% sequence similarity to obtain the nonredundant protein sequence set.

#### Protein functional annotation

The nonredundant protein sequence set was compared with the protein database to annotate the gene function in each sample. The databases to provide the functional annotations are as follows:

**KEGG** (Kyoto Encyclopedia of Genes and Genomes, http://www.genome.jp/kegg/)^24^;

**EggNOG** (Evolutionary Genealogy of Genes: Non-supervised Orthologous Groups, http://eggnogdb.embl.de/)^25^;

**CAZy** (Carbohydrate-Active enZymes Database, http://www.cazy.org/)^26^

**QIIME** (Quantitative Insights Into Microbial Ecology)^27^ was used to obtain the relative abundance distribution of each sample that correspond to each functional level in each database.

In addition, the common and unique KO (KEGG Orthology) of the two samples were obtained by comparison, and the common/unique KEGG metabolic pathway was analyzed. The SciPy library of Python software was used to compare and statistically test the KO functional groups with different abundance levels through the hypergeometric distribution, and the enrichment analysis of KEGG metabolic pathways was conducted.

### Analysis

The influent and effluent samples were collected daily and immediately analyzed. COD was analyzed using a COD quick-analysis apparatus (Lian-hua Tech. Co., Ltd., 5B-1, China). The concentration of nitrogen compounds and total phosphorus (TP) were determined by reference to the standard method^28,29^. The temperature was measured by thermometers. The pH was measured by a digital portable pH meter, and the DO (dissolved oxygen) was measured by a digital portable DO meter (YSI, Model 55, USA). The induction intensity of the magnetic field was measured by a digital tesla meter (CESTSEN, HT20, China).

## Results

### Long-term effects of magnetic field on performance of SBRs

**Figure 1:**
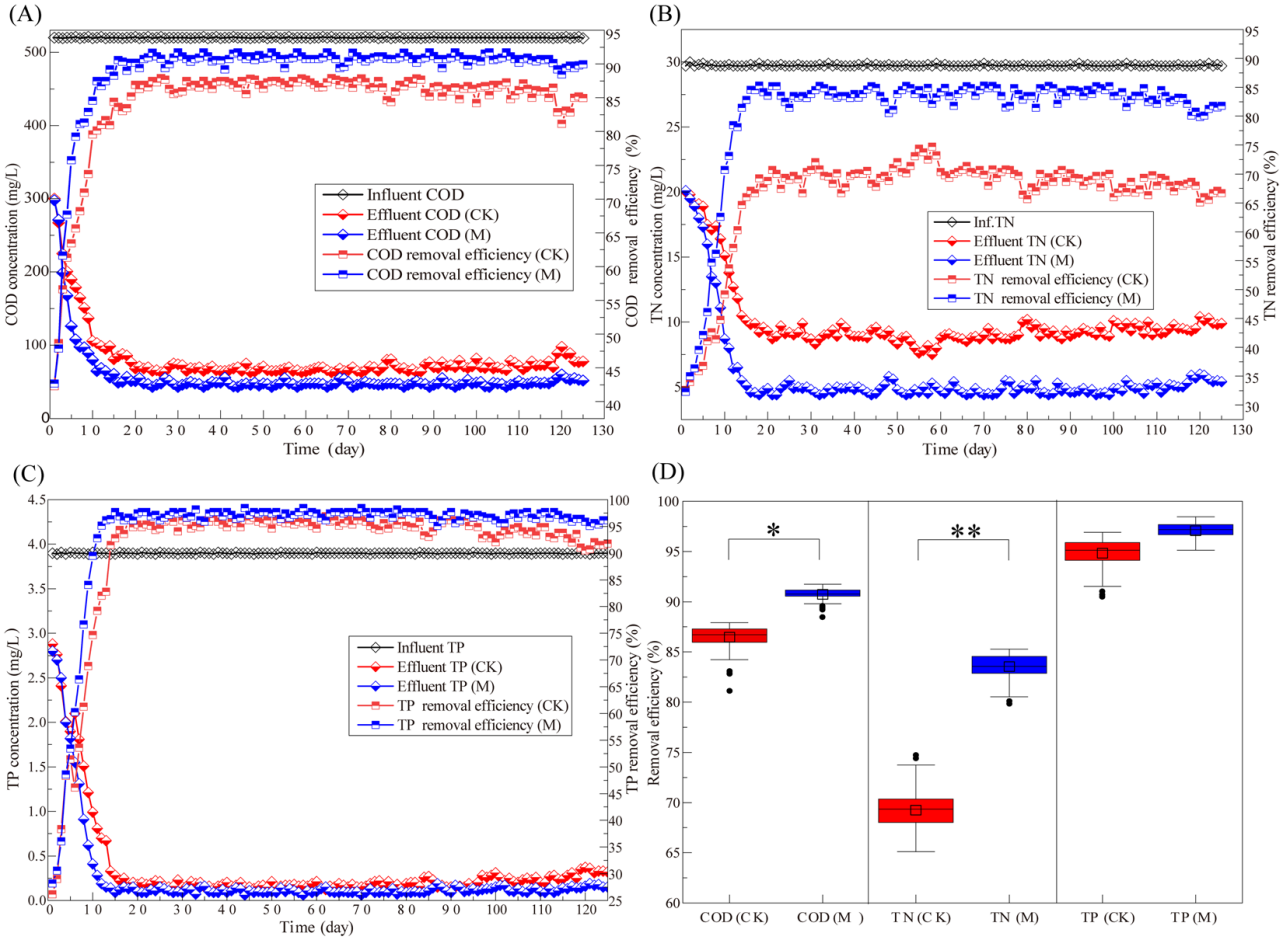
Long-term treatment performance of wastewater with (M) and without (CK) the application of a magnetic field: (**A**) changes in COD concentration and removal rate; (**B**) changes in TN concentration and removal rate; (**C**) changes in TP concentration and removal rate; and (**D**) comparison of the pollution removal effect.

**Figure 2:**
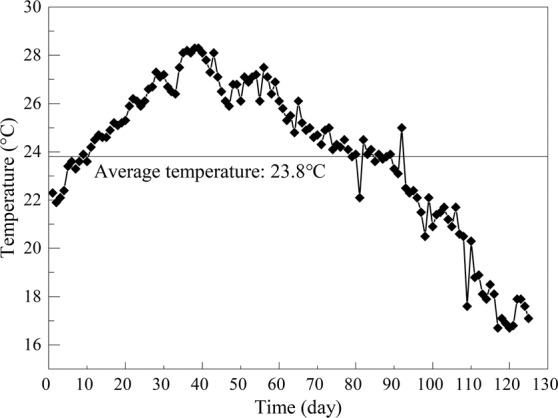
Changes in ambient temperature during the experiment.

After 125 days of long-term laboratory operation, the performance of the reactors with and without the application of a magnetic field was recorded as shown in Fig. 1. In the experimental stage, the temperature (Fig. 2) varied in the range of 16.7 °C-28.3 °C (23.8 °C on average), and the average influent COD, TN, and TP were 520 mg·L^−1^, 29.7 mg·L^−1^ and 3.9 mg·L^−1^, respectively. With the acclimation of sludge, the wastewater treatment process ran smoothly after 16 days for M and 20 days for CK. As shown in Fig. 1(d), the removal ability presented an obvious difference between the conditions with and without magnetic field, which suggests that the magnetic field plays an important role in the removal of pollutants. The final removal rates of COD, TN and TP at 70 mT were 89.9%, 81.8% and 96.1%, respectively. Compared with the control group, the final removal rates increased by 5%, 15.2% and 4.3%, respectively, which indicates a higher removal efficiency of the magnetic field. In addition, after the operation was stable (after 20 days), the ranges of COD, TN, and TP removal rates with magnetic field were 3.29%, 5.41%, and 3.35%, respectively, which were lower than the control group (COD: 6.75%, TN: 9.65%, TP: 6.41%). Therefore, though the temperature changed with the seasons during the experiment, the wastewater treatment performance was more stable with the 70-mT magnetic field. In summary, the application of a magnetic field to the SBR reactor can effectively shorten the time from start-up to stable operation of the SBR process and improve the operation performance of the reactor.

### Overview of metagenome sequencing data

The sludge samples were collected on day 125 at the aeration stage of CK and M reactors in triplicate, respectively, and finally mixed evenly to conduct DNA extraction. The metagenome sequencing of the activated sludge samples with and without magnetic field resulted in total of 72,952,276 and 104,261,932 raw reads, respectively. Clean reads were obtained by filtering the sequence data, which was 65,438,191 and 94,231,934, respectively. All clean reads were de novo assembled by using SOAPec (v2.01) (the two samples were merged for greater stability). As a result, 444,274 contigs with a minimum length of 200 bp and a maximum contig length of 669,717 bp were obtained. As well, the clean reads assembly generated 67,707 scaffolds with a minimum length of 200 bp and a maximum contig length of 739,536 bp (Table 1).

**Table 1:** Assembly and sequencing statistics for the activated sludge samples.

Parameter	CK	M
Raw reads	104,261,932	72,952,276
Raw bases	15,743,551,732	11,015,793,676
Clean reads	94,231,934	65,438,191
Clean bases	14,098,350,576	9,791,938,998
**Metagenomic sequence assembling**	**Contigs**	**Scaffolds**
Minimum sequence length (bp)	200	200
Maximum sequence length (bp)	669,717	739,536
N50 length (bp)	1,902	2,050
Total sequences	444,274	67,707
Macronomic length (bp)	532,318,741	530,334,523
GC base count	300,608,981	299,397,251
Sequences of length > 1 kb	119,179	116.148

### Functional gene classification

To explain the effects of the magnetic field on sludge at the gene level, species and abundances of functional genes were annotated based on the KEGG, eggNOG, and CAZy databases.

The metabolic pathway analysis database is the core of the KEGG database. By comparing the protein sequences with the KEGG metabolic pathway database, the genes predicted by the macrogenome were annotated and classified according to the metabolic pathways. Unigenes were assigned to 40 Level 2 KEGG items, which were summarized in six KEGG categories: cellular processes, environmental information processing, genetic information processing, human diseases, metabolism, and organismal systems.

**Figure 3:**
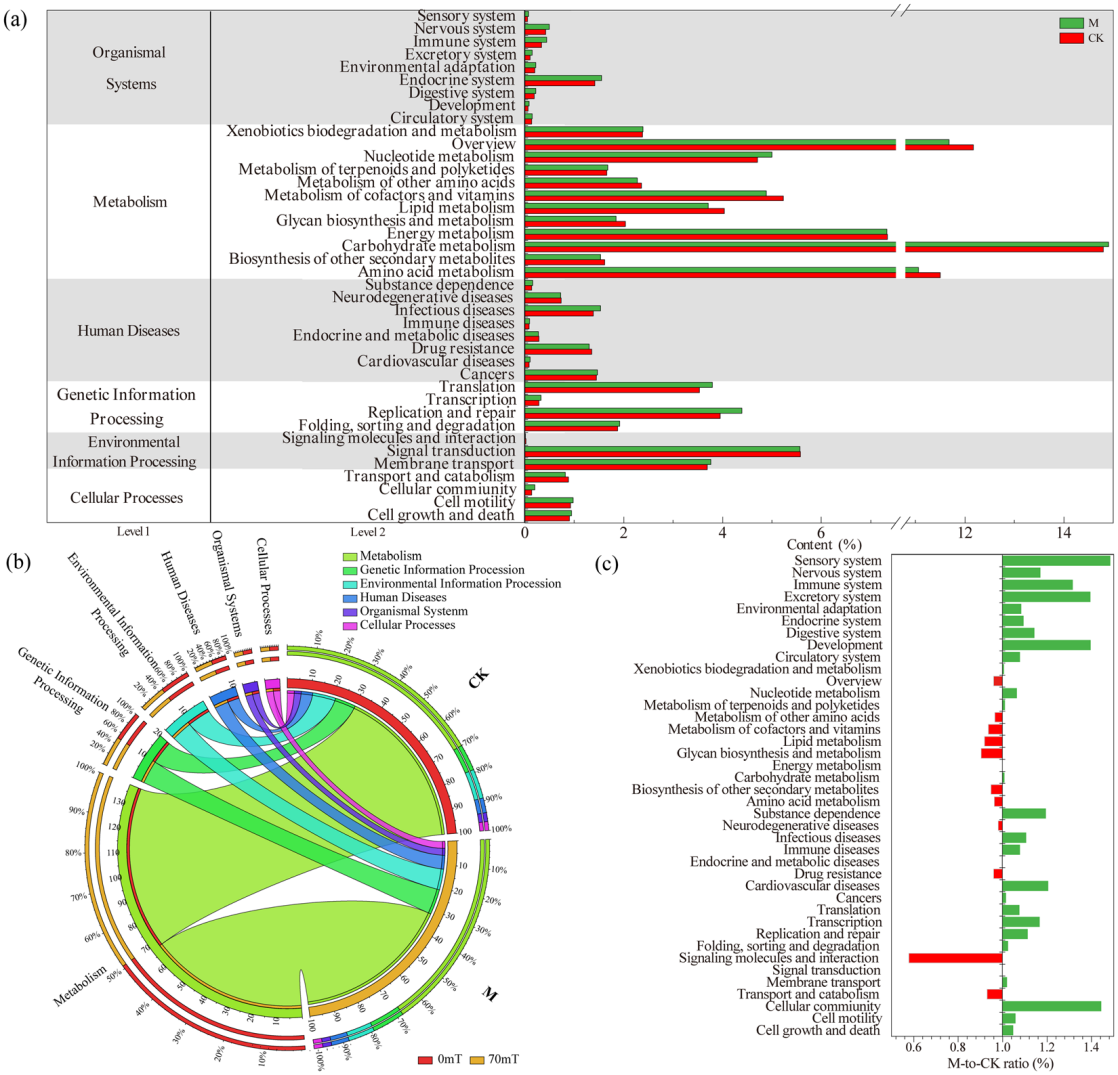
Distribution of the KEGG classification in the activated sludge macrogenome: (**a**) composition and abundance of six KEGG functional genes in the macrogenome of activated sludge; (**b**) content of KEGG level-1 functional genes in the macrogenome of activated sludge; and (**c**) the ratios of unigene contents in M to CK.

As shown in Fig. 3(b), the relationships between samples and functional gene categories are shown in Circos figures. Among all six KEGG categories, the most abundant unigenes in the two activated sludge macrogenomes was metabolism, followed by genetic information processing, environmental information processing, human diseases, organismal systems and cellular processes. The order of the contents of the six KEGG categories in the two groups of activated sludge macrogenomes was identical, i.e., the order of the six KEGG functional genes in the macrogenome was not changed by the action of the 70-mT magnetic field on the activated sludge. In addition, the functional gene contents of Cellular Processes, Environmental Information Processing, Genetic Information Processing, Human Diseases, and Organismal Systems were higher with the magnetic field (2.94%, 9.34%, 10.43%, 5.65%, and 3.37%, respectively) than without (2.84%, 9.28%, 9.63%, 5.50%, and 2.92%, respectively). But functional genes related to Metabolism decreased with the addition of the 70-mT magnetic field (69.82% to 68.27%).

In the KEGG subcategories, the topmost unigene in the two activated sludge macrogenomes was carbohydrate metabolism (0 T: 14.80%; 70 mT: 14.91%) in metabolism, followed by overview (0 T: 12.16%; 70 mT: 11.67%), amino acid metabolism (0 T: 11.50%; 70 mT: 11.06%), energy metabolism (0 T: 7.34%; 70 mT: 7.33%), and signal transduction (0 T: 5.57%; 70 mT: 5.56%); the contents of the five rich functional genes were sequenced in the same order (Fig. 3a). In addition, there were six KEGG subcategories, which were more than 1.2 times more abundant in the 70-mT magnetic field than in the untreated field, including sensory system, cellular community, development, excretory system, immune system, and cardiovascular diseases. However, the content of Signaling molecules and interaction related genes in the macrogenome of activated sludge under 70-mT (0.011%) was 0.58 times lower than that of the unmagnetized activated sludge (0.020%) (Fig. 3c). Additionally, 370 KEGG level 3 metabolic pathways were annotated. Among them are pathways that play a major role in the pollutant degradation in sewage treatment, including nitrogen metabolism (0 T: 0.8379%; 70mT: 0.8600%), starch and sucrose metabolism (0 T: 1.2071%; 70 mT: 1.3276%), sulfur metabolism (0 T: 0.7433%; 70 mT: 0.7299%) related functional genes in the energy metabolism category, and carbon metabolism (0 T: 4.4722%; 70 mT: 4.3900%) related functional genes in the Overview category.

**Figure 4:**
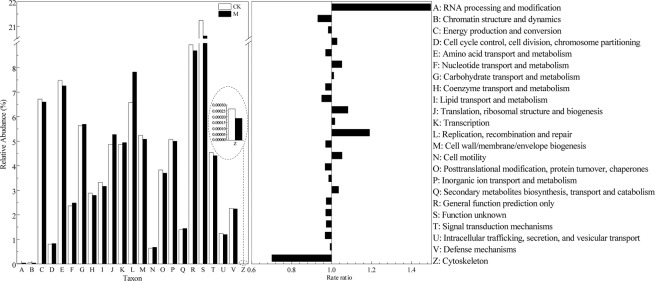
Distribution and comparison of the eggNOG categories.

EggNOG mainly consists of information storage and processing, cellular processes and signaling, metabolism, and poorly characterized. Orthologous groups, functional annotations and protein sequences are available from eggNOG^25^. For the eggNOG classification, unigenes were categorized into 25 categories. At the top (Fig. 4), not considering the unknown function category, the clusters included R (general function prediction only), E (amino acid transport and metabolism), C (energy production and conversion), G (Carbohydrate transport and metabolism), and L (replication, recombination and repair). Most of these categories are related to the metabolism and information storage and processing. Additionally, the functional gene abundance shows that A (RNA processing and modification), L (Replication, recombination and repair), J (Translation, ribosomal structure and biogenesis), and N (Cell motility) enriched in M compared with CK. Specifically, as shown in Fig. 4, RNA processing and modification, and Replication, recombination and repair relative contents under the magnetic field were approximately 1.49 and 1.19 times that of the control group, respectively. The interference of magnetic field on the category Z (Cytoskeleton) was obvious, and the content of cytoskeleton was reduced by 30%.

**Figure 5:**
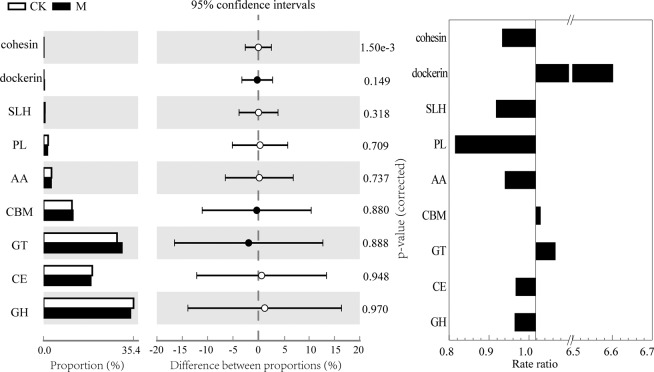
Distribution differences among the CAZy classifications.

The CAZy database focuses on carbohydrate active enzymes that can degrade, modify, or generate Glycosidic bonds. These carbohydrate active enzymes are divided into six major protein functional modules^26^: glycoside hydrolases (GH), glycosyl transferases (GT), polysaccharide lyases (PL), esterases (CE), auxiliary activities (AA), carbohydrate-binding modules (CBM). As shown in Fig. 5, GH, GT, and CE were the most abundant carbohydrate active enzymes in all activated sludge macrobolomics. Among all categories, the magnetic field greatly interferenced the PL, whose relative content decreased the most. Meanwhile, the abundance of GT under the action of the magnetic field was higher than that without a magnetic field. The increase in GT will benefit the entire energy supply pathway^30^.

Based on the relative abundance of the underlying functional groups annotated in the functional databases of each sample, the differences of each functional group (KO, NOG and CAZy protein families) between the two samples were compared. When the difference between the two samples is more than 2 times (|Log_2_ (Fold_change_value)| > 1) and P < 0.05, the functional category is considered to have a significant difference. According to Table 2, there were 3368, 24055 and 62 groups with significant differences in KO, NOG and CAZy in the two samples.

**Table 2:** Statistical table for the abundance difference analysis.

Group	KO	eggNOG	CAZy
CK-M	3,368	24,055	62

### The main pathway influenced by magnetic field

**Figure 6:**
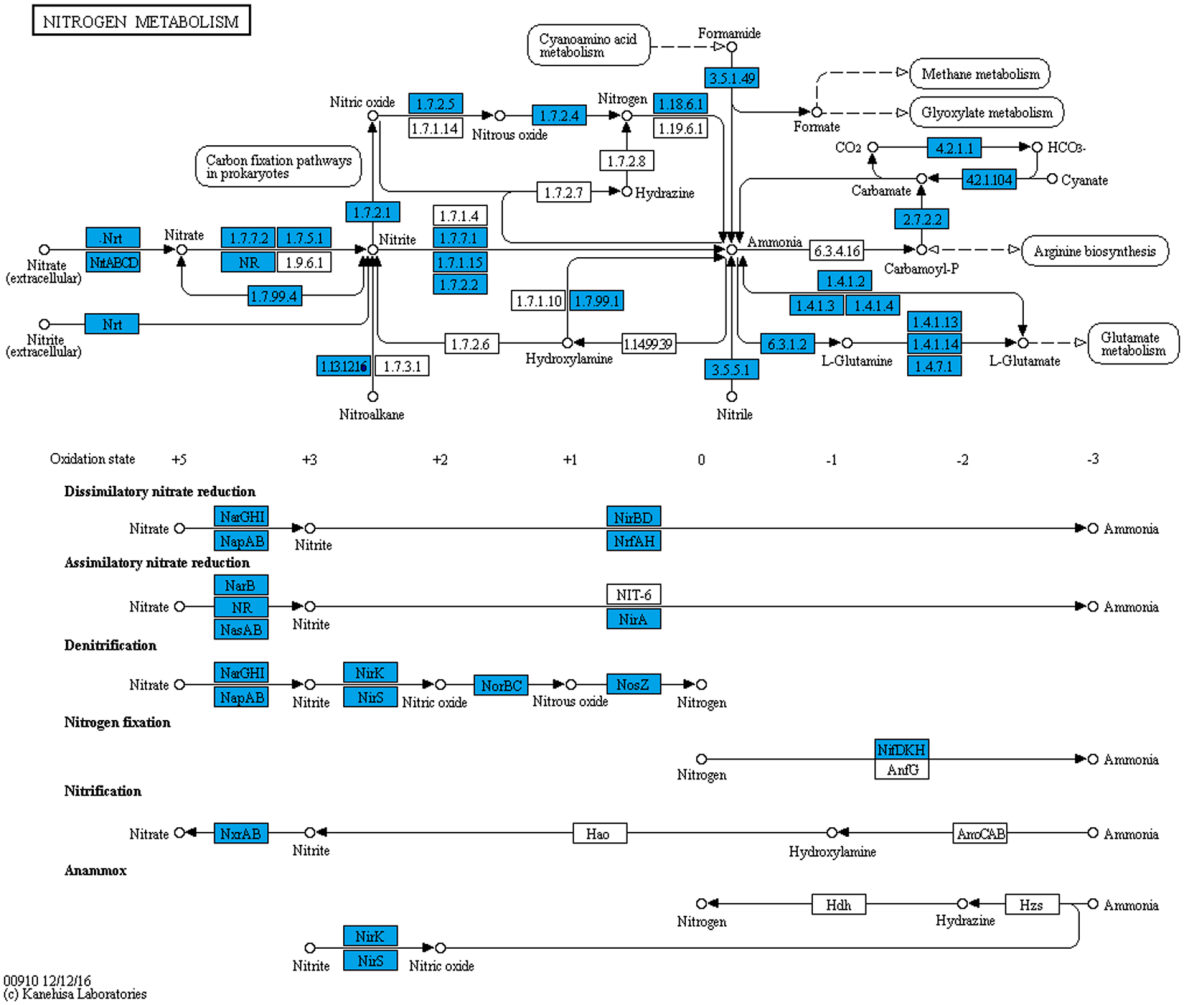
Common KEGG metabolic pathway.

Denitrification is an important function of activated sludge, and the removal of TN is closely related to the functional genes in the nitrogen metabolism in activated sludge macronomic. Since the 70-mT magnetic field significantly promoted the removal of TN, we focused on the analysis of the functional genes and pathways related to nitrogen metabolism of KEGG. According to the KO00910 pathway map of nitrogen metabolism (Fig. 6), the biochemical process of nitrogen cycling is a complex interaction among many microorganisms that catalyze different reactions, where nitrogen has different oxidation states from +5 valence in nitrate to -3 valence in ammonia. The nitrogen cycle mainly involves nitrogen fixation, assimilatory nitrate reduction and dissimilatory nitrate reduction, denitrification, nitrification, anaerobic ammonia oxidation (anammox) and others. As shown in Fig. 6, all detected KO terms were marked in blue which indicates that they simultaneously appeared, and there was no unique KO in the two samples, which suggests that the protein types of genes related to the nitrogen metabolism in the activated sludge macromonome did not change under the 70 mT magnetic field.

From the enrichment analysis (Fig. S1), the magnetic field seriously impacted ferredoxin-nitrate reductase [EC:1.7.7.2], ferredoxin-nitrite reductase [EC:1.7.7.1], nitrilase [EC:3.5.5.1], glutamate dehydrogenase [EC:1.4.1.2], and carbonic anhydrase [EC:4.2.1.1]. Among them, ferredoxin-nitrate reductase [EC:1.7.7.2] and ferredoxin-nitrite reductase [EC:1.7.7.1] are the major enzymes in the assimilatory nitrate reduction process, which can enhance the ability to convert nitrate into ammonia^18^. The 70-mT magnetic field reduced the contents of ferredoxin-reductase [EC:1.7.7.2] and ferredoxin-nitrite reductase [EC:1.7.7.1], which inhibited the assimilation nitrate reduction reaction. The inhibition of the ammonification process, will reduce the production of ammonia nitrogen, and the accumulation of nitrate and a small amount of nitrite under certain conditions will also promote the denitrification reaction. This result is consistent with the effluent concentration of total nitrogen.

## Discussion

The exploitation of new methods to enhance the wastewater treatment capacity for more severe pollution and increasingly stringent requirements on sewage discharge has received much attention over the past decades. Studies have accumulated over the years to emphasize the importance of magnetic fields, which are used as an effective choice to improve the wastewater treatment performance. Previous studies^12^ have confirmed that medium- and low- intensity magnetic fields have a positively effect on the wastewater treatment system, and the best effect is achieved at approximately 70 mT. Therefore, the optimum magnetic field intensity of 70 mT was selected to explore its long-term effects on the functional genes of activated sludge microorganisms in the present study.

The data showed that magnetic fields improved the ability of the activated sludge to remove COD, TN, and TP by 5%, 15.2% and 4.3%, respectively, at 70 mT compared with that of the reactor with no magnetic field. Among them, the TN elimination was the most effective for the reactor with magnetic field. Furthermore, the pollutant removal performance remained more stable during the long-term operation despite the low temperature in comparison with the control. The reactor without the magnetic field was more susceptible to the temperature decrease. Those results prove that the magnetic field enhanced the ability and stability of the wastewater treatment as the inclusion of the 70 mT magnetic field resulted in better performance than that observed for the CK group.

However, the role of the magnetic field and its effect on functioning of biological organisms remain insufficiently understood and are being actively studied^31^. Functional annotation and classification were performed to expound the activated sludge metagenome in this study. For the eggNOG classification, categories such as RNA processing and modification, and replication, recombination and repair were more abundant in the reactors with a magnetic field when compared with CK, which suggests that functional genes related to information storage and processing were enhanced by the external magnetic field. The functional genes abundance annotated based on the CAZy database shows that the magnetic field greatly interferences the PL, which can depolymerize certain acidic polysaccharides through an eliminative mechanism^32^. Meanwhile, the abundance of GT under 70 mT magnetic field is 6% higher than that without a magnetic field, which has proven to be beneficial to the entire energy supply pathway^30^. The difference in content and distribution of CAZy and NOG in the macrogenome of activated sludge indicates that the magnetic field can affect the wastewater treatment performance of activated sludge by changing the abundance of functional genes.

To investigate the metabolic functions of the detected unigenes, a pathway-based analysis, KEGG, was used. In the KEGG categories, Sensory system, Cellular community, Development, Excretory system, Immune system, and Cardiovascular diseases represented the major subcategories significantly increased under the 70-mT magnetic field. However, the content of signaling molecules and interaction-related genes were significantly inhibited by the 70-mT magnetic field. Since the long-term operation of SBRs revealed that the 70-mT magnetic field significantly promoted the removal of TN, the functional genes and pathways related to nitrogen metabolism were analyzed. The common/specific KEGG metabolic pathway analysis show that protein types deriving from genes related to the nitrogen metabolism were detected in both reactors. From the enrichment analysis, enzymes such as ferredoxin-nitrate reductase [EC:1.7.7.2], ferredoxin-nitrite reductase [EC:1.7.7.1], nitrilase [EC:3.5.5.1], glutamate dehydrogenase [EC:1.4.1.2], and carbonic anhydrase [EC:4.2.1.1] were significantly inhibited by the magnetic field. These enzymes are regulated by NarB and NirA. The inhibition of ferredoxin reductase and ferredoxin nitrite reductase represents the inhibition of assimilation nitrate reduction reaction, which belongs to the ammonification process; thus the production of ammonia nitrogen will be reduced, and ammonia oxidation will be more obvious. This study has a similar conclusion to others^14^, in which the weak magnetic field positively affects the activity of AOB (ammonia oxidizing bacteria). This result is consistent with the effluent concentration of total nitrogen.

In the present study, due to the different emphasis of annotation, the eggNOG, CAZy and KEGG databases were selected to annotate the activated sludge macrogenome. KEGG focuses on the annotation of metabolic pathways, eggNOG focuses on the annotation of direct homologous compounds, and CAZy focuses on the annotation of carbohydrate active enzymes that degrade, modify, or generate glycosides. Inevitably, the annotated genes may be overlapped. However, in general, the obvious improvement of magnetic field on the performance of activated sludge in treating nitrogen is mainly related to the nitrogen metabolism pathway in the KEGG database.

## Conclusions

The pollutant removal performance and biological effects due to a 70-mT magnetic field on the active sludge of long-term running SBRs were investigated at ambient temperature in this study. Compared to the absence of a magnetic field, the SBR reactor took less time from start-up to stable operation with the addition of the 70-mT magnetic field. For over 125 days, the pollutant removal for the reactor in which the activated sludge was exposed to the magnetic field was higher than the control, and the elimination of TN was the most effective with the magnetic field. The functional genes and pathways related to the nitrogen metabolism were analyzed based on the KEGG database, which indicated that all enzymes existed in both reactors; the inhibition of NarB and NirA by the magnetic field was related to the improved nitrogen removal capacity. The unigenes related to information storage and processing were enhanced by the external magnetic field. Additionally, glycosyl transferases were more abundant in the reactors with a magnetic field compared with CK, which has proven to be beneficial to the entire energy supply pathway. The magnetic field can affect the wastewater treatment performance of activated sludge by changing the abundance of functional genes.

## Supplementary information

Supplementary information.
