# Supplementary material for: Effects of an external magnetic field on microbial functional genes and metabolism of activated sludge based on metagenomic sequencing
Source: Sci Rep. 2020 Jun 1;10:8818. doi: 10.1038/s41598-020-65795-3 (PMC7264255; doi:10.1038/s41598-020-65795-3)
Supplement: Supplementary file 1 — Supplementary information. [file 41598_2020_65795_MOESM1_ESM.doc]

**Effects of an external magnetic field on microbial functional genes and metabolism of activated sludge based on metagenomic sequencing**

Shuying Geng^1, 2^, Weizhang Fu^1^, *, Weifeng Chen^1^, *, Shulian Zheng^3^, Qi Gao^4^, Jing Wang^1^, Xiaohong Ge^1^

^1^ College of Resources and Environment, Shandong Agricultural University, Taian 271018, China

^2^ College of Water Sciences, Beijing Normal University, Beijing 100875, China

^3^ Taian Chuanyuan Environmental Protection Equipment Co., Ltd, Taian 271000, China

^4^ College of Food Science and Engineering, Shandong Agriculture and Engineering University, Dezhou 251100, China

* **Co-corresponding author:**

Weizhang Fu; Phone No.: +8613905383700; E-mail: sdaufwz@sdau.edu.cn

Weifeng Chen; Tel: +86 (0538) 8241371; E-mail: chwf@sdau.edu.cn


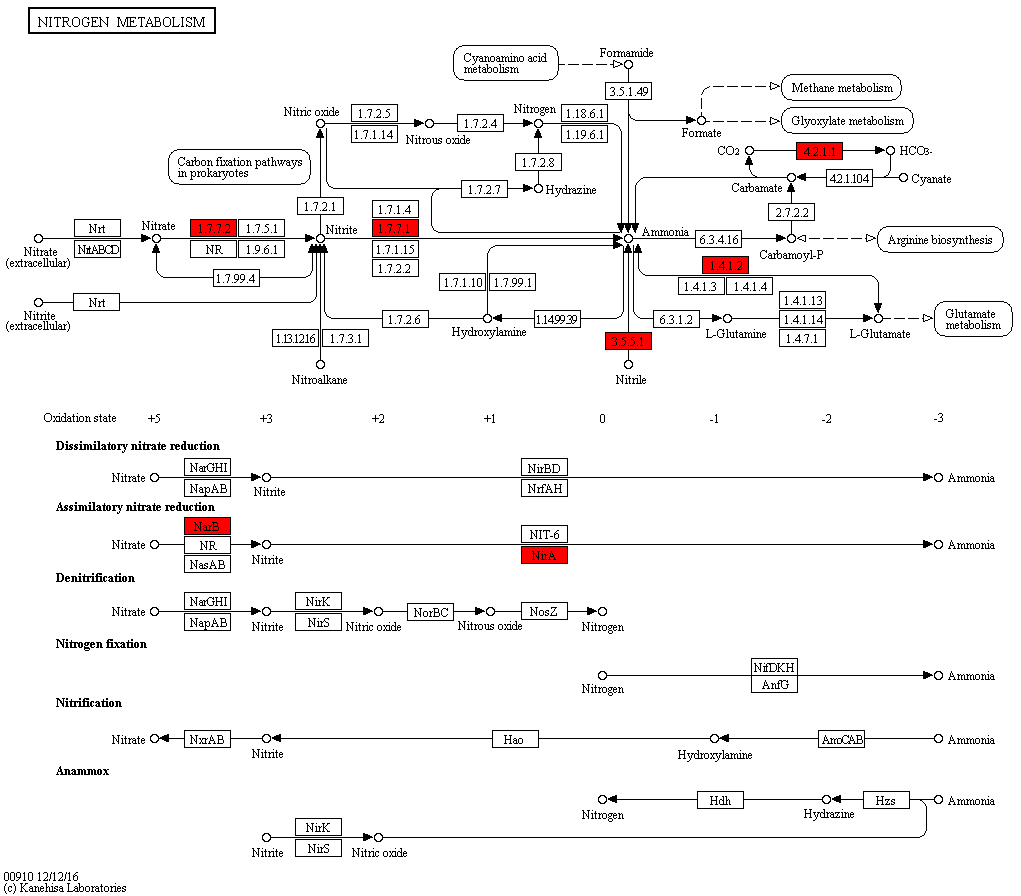


**Figure S1** KEGG metabolic pathway enrichment map
